# Supplementary material for: Hardening in Au-Ag nanoboxes from stacking fault-dislocation interactions
Source: Nat Commun. 2020 Jun 10;11:2923. doi: 10.1038/s41467-020-16760-1 (PMC7287112; doi:10.1038/s41467-020-16760-1)
Supplement: Supplementary file 1 — Supplementary Information [file 41467_2020_16760_MOESM1_ESM.pdf]

Supplementary Information for

Hardening in Au-Ag Nanoboxes from Stacking Fault-Dislocation Interactions

Radhika P. Patil[1], David Doan[1], Zachary H. Aitken[3], Shuai Chen[3], Mehrdad T. Kiani[2], Christopher M. Barr[4], Khalid Hattar[4], Yong-Wei Zhang[3], X. Wendy Gu*[1]

[1]Department of Mechanical Engineering, Stanford University, Stanford, CA 94305, United States

[2]Department of Materials Science and Engineering, Stanford University, Stanford, CA 94305, United States

[3]Institute of High Performance Computing, A*STAR, 1 Fusionopolis Way, #16-16 Connexis, 138632 Singapore

[4]Materials, Physical, and Chemical Sciences, Sandia National Laboratories, Albuquerque, NM, 87185, USA

*Email: xwgu@stanford.edu

Supplementary Methods – MD simulation

We investigated the effect of sample geometry on the deformation response of the simulated nanoboxes by preparing geometries with wall thicknesses of 7 nm, 3.125 nm and 2 nm, translating to aspect ratios of 1:7 (similar geometry as the experimental nanoboxes), 1:16, and 1:25 for an overall size of 50 nm. The samples were loaded as shown in Supplementary Fig 2. The samples were loaded at applied force rates of 10^8, 10^7, 10^6, and 10^5 μN/s (Supplementary Fig. 3), and the results of the 10^5 μN/s was determined to be the most realistic. The stress-strain data of all samples can be qualitatively described as following 3 stages of deformation. There is an initial elastic segment followed by a local peak and stress drop which corresponds to initial sample failure. This peak occurs at 3.81%, 4.87%, and 4.94% strain and the peak stress is 585 MPa, 245 MPa, and 143 MPa, for the samples with 1:7, 1:16, and 1:25 aspect ratio, respectively. All samples then undergo further plastic deformation which is characterized as a linear regime extending from 8.5% to 48.8% in the 1:7 sample, from 6.2% to 66.3% in the 1:16 sample, and from 8.0% to 73.8% in the 1:25 sample. The slope during this linear regime is 1026 MPa, 612 MPa, and 50 MPa, for the 1:7, 1:16, and 1:25 samples respectively. The final stage of deformation in the stress-strain data is characterized by a rapid increase in the stress. Atomic output indicates that this regime is associated with contact between different sides of the nanobox.

Supplementary Methods – FEM simulation

We investigated the effect of the nanobox geometry on the hardening response, given an elastic perfectly plastic material model. The dimensions of the simulated nanobox were derived from the average dimensions of the experimental samples: side length of 139 nm and wall thickness of 18 nm. For the main text, an isotropic model with Young's modulus and Possion's ratio of 47

GPa and 0.35 are used, respectively. An anisotropic, elastic perfectly plastic model was also used to see the effects of anisotropy, with the elastic stiffness tensor populated as shown (c_{11} = 124 GPa, c_{12} = 93.4 GPa, c_{44} = 46.1 GPa):

$$\boldsymbol{C} = \begin{bmatrix} 124\ GPa & 93.4\ GPa & 93.4\ GPa & & & \\ & 124\ GPa & 93.4\ GPa & & & \\ & & 124\ GPa & & & \\ & & & 46.1\ GPa & & \\ & & & & 46.1\ GPa & \\ & & & & & 46.1\ GPa \end{bmatrix}$$

A yield stress of 600 MPa was used, which is derived from experimental Ag <100> compressions. The simulated nanoboxes were then compressed along a principal direction both show similar results with a yield of ~230 MPa and slight strain softening after ~8% strain.

In order to compensate for possible strain softening, a Riks (or arc length) method was utilized. To ensure that the softening response that we observe from the simulation was not numerical error from the Riks method, we ran a control case on a solid nanocube. The solid nanocube engineering stress-strain plot showed some strain hardening. The strain hardening, which was expected due to the area increase of the solid nanocube, shows that the softening in our nanobox is not purely due to numerical error from the Riks method.

Supplementary Figures

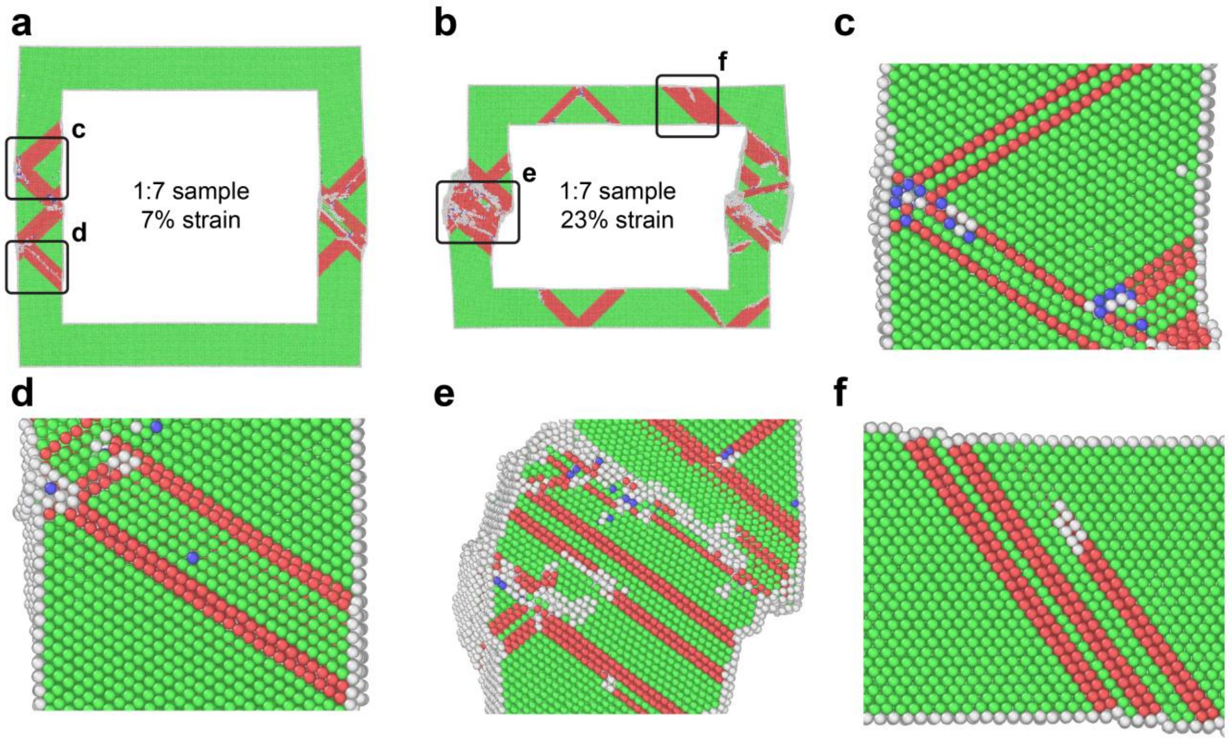

Supplementary Figure 1. Cross-sectional view of 1:7 smooth nanobox sample (ratio of wall thickness to length). Nanobox deformed at **a** 7% strain and **b** 23% strain showing nucleated stacking faults. Green atoms have FCC coordination and red atoms are stacking faults. **c,d,e,f** Enlarged regions marked by corresponding alphabet in **a,b**

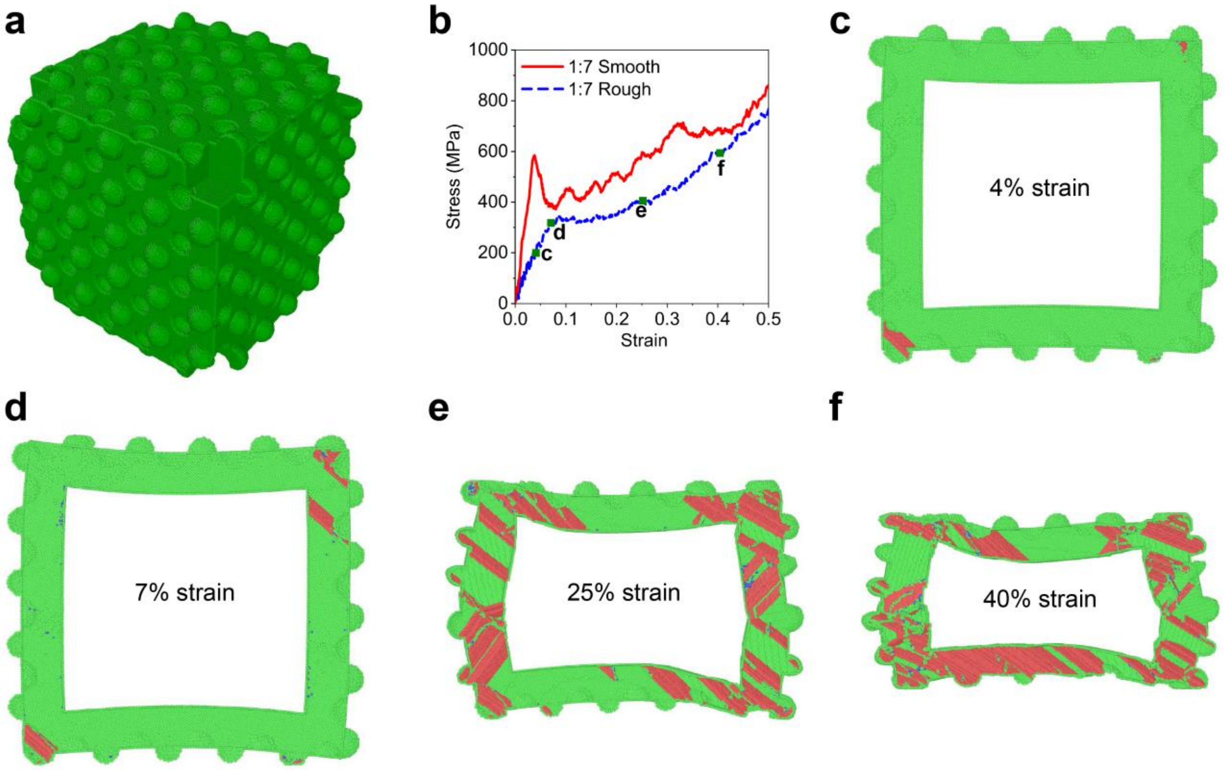

Supplementary Figure 2. MD simulations of Ag nanobox with rough protrusions and indents. **a** The rough nanobox sample. **b** Comparison between the generated stress-strain data of smooth and rough nanoboxes. Cross-sectional view of 1:7 rough sample (ratio of wall thickness to length) at **c** 4% strain, **d** 7% strain, **e** 25% strain, and **f** 40% strain. Green atoms have FCC coordination and red atoms are stacking faults.

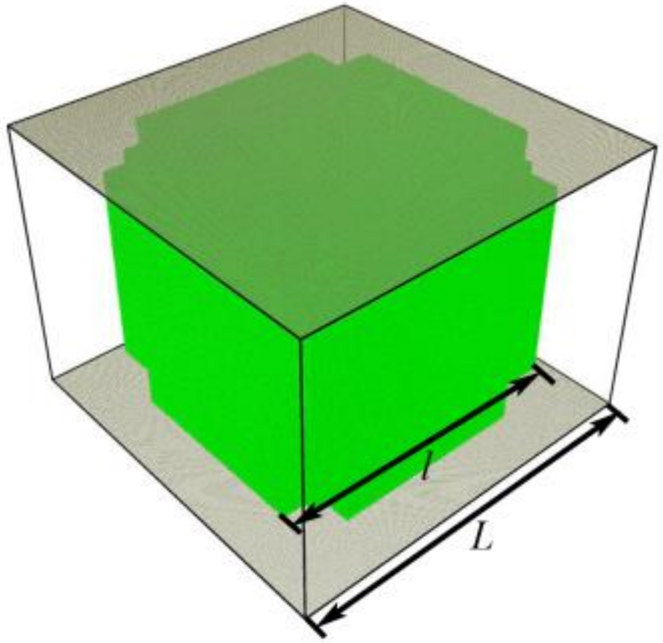

Supplementary Figure 3. Schematic of MD simulation showing the Ag nanobox with side length *l* (green atoms) and the compressing plates above and below the nanobox with side length *L* (black atoms).

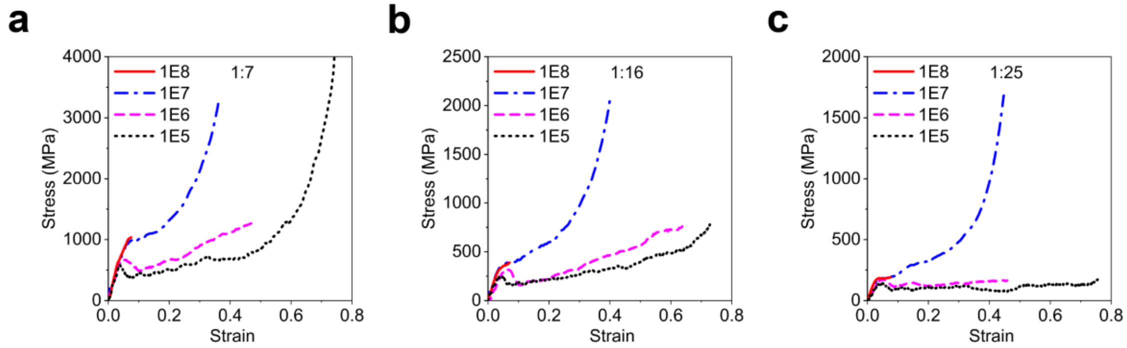

Supplementary Figure 4. MD simulated stress-strain data at applied force rates of 10^8, 10^7, 10^6, and 10^5 µN/s. Plots for samples with A) 1:7 aspect ratio, B) 1:16 aspect ratio, and C) 1:25 aspect ratio.

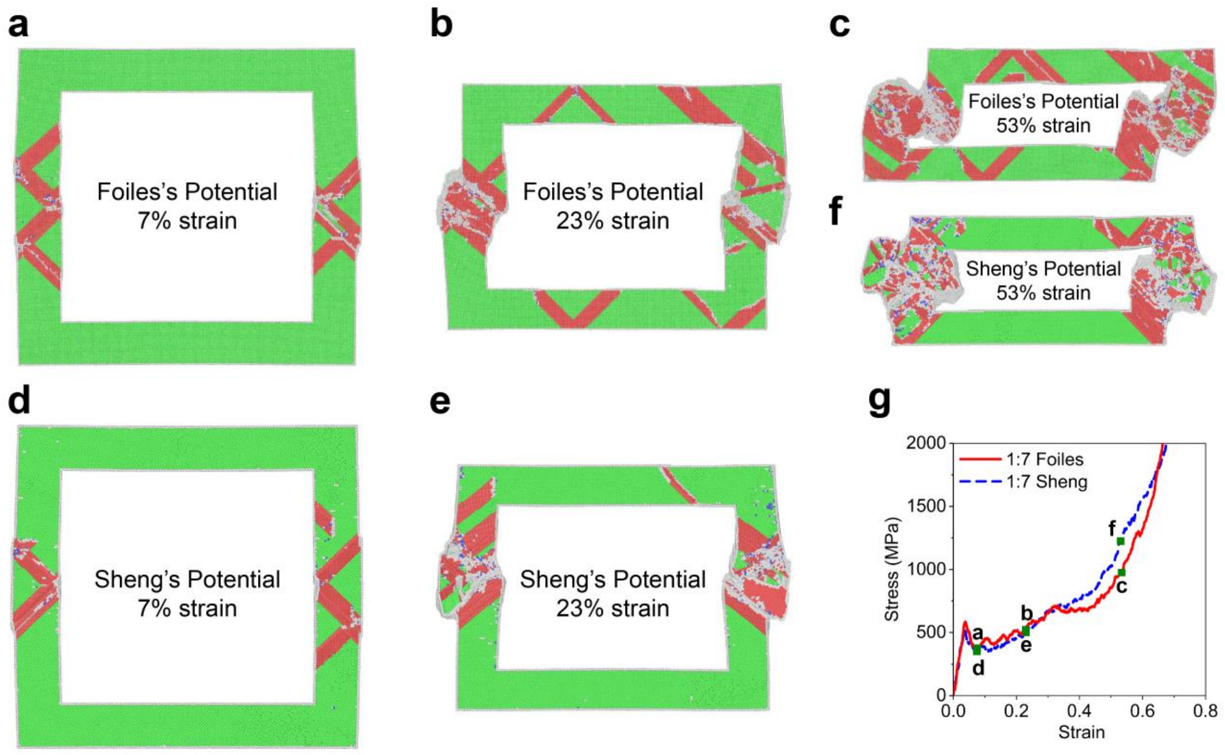

Supplementary Figure 5. MD simulations with the Foiles and Sheng potentials.[1,2] Nanoboxes compressed to A) 7%, B) 23%, C) 53% strain using the Foiles potential. Nanoboxes compressed to D) 7%, E) 23%, F) 53% strain using the Sheng potential. G) The stress-strain curves for the two potentials.

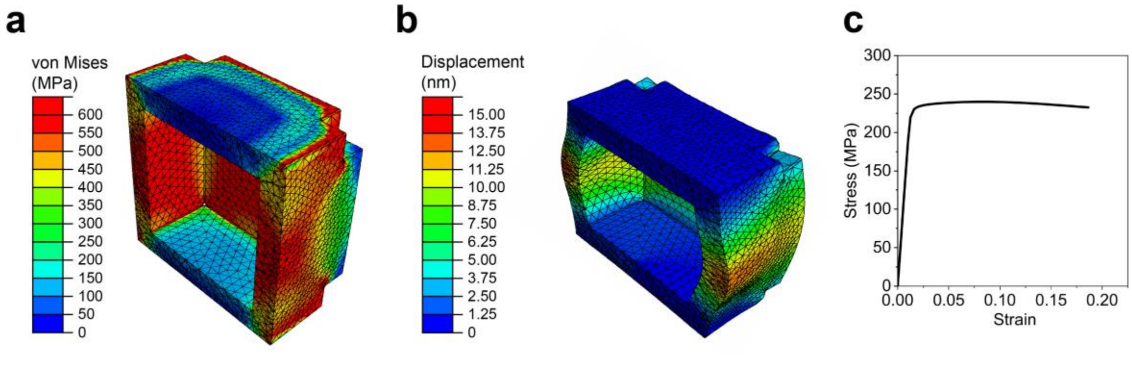

Supplementary Figure 6. FEM simulation of a smooth nanobox using an anisotropic model. Cross-sectional view of **a** von Mises stress at ~1.5% strain, **b** radial displacement at ~18% strain, and **c** corresponding engineering stress-strain curve.

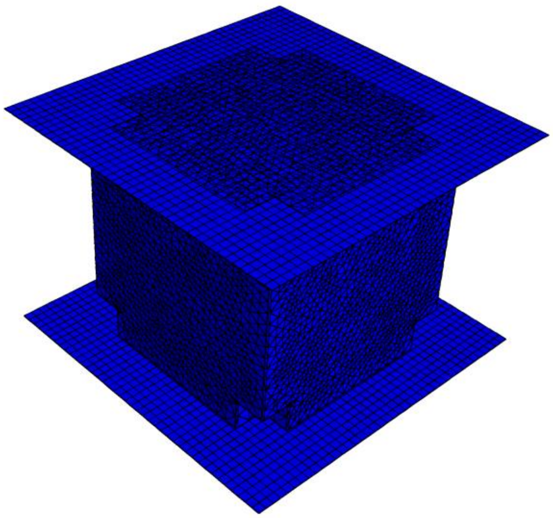

Supplementary Figure 7. FEM simulation showing the Ag nanobox and the compressing plates above and below the nanobox.

Supplementary References

1. Foiles, S. M., Baskes, M. I. & Daw, M. S. Embedded-atom-method functions for the fcc metals Cu, Ag, Au, Ni, Pd, Pt, and their alloys. *Phys. Rev. B* **33**, 7983–7991 (1986).

2. Sheng, H. W., Kramer, M. J., Cadien, A., Fujita, T. & Chen, M. W. Highly optimized embedded-atom-method potentials for fourteen fcc metals. *Phys. Rev. B* **83**, 134118 (2011).
