# Supplementary material for: Hardening in Au-Ag nanoboxes from stacking fault-dislocation interactions
Source: Nat Commun. 2020 Jun 10;11:2923. doi: 10.1038/s41467-020-16760-1 (PMC7287112; doi:10.1038/s41467-020-16760-1)
Supplement: Supplementary file 3 — Description of Additional Supplementary Files [file 41467_2020_16760_MOESM3_ESM.docx]

Description of Additional Supplementary Files

**Title:** Supplementary Movie 1.

**Description:** In-situ TEM compression test on smooth Au-Ag nanobox.
